# Chronic inflammatory bowel diseases and sexuality: Inevitable disorders?

**DOI:** 10.1192/j.eurpsy.2021.1473

**Published:** 2021-08-13

**Authors:** N. Charfi, A. Guermazi, F. Guermazi, S. Omri, N. Smaoui, R. Feki, J. Ben Thabet, L. Zouari, M. Maalej Bouali, N. Tahri, M. Boudabous, M. Maalej

**Affiliations:** 1 Psychiatry C Department, Hedi chaker University hospital, sfax, Tunisia; 2 Gastroenterology Department, Hedi chaker University hospital, sfax, Tunisia

**Keywords:** Chronic Inflammatory Bowel Diseases, Sexual Dysfunction, quality of sexual life

## Abstract

**Introduction:**

Improving the quality of sexual life of patients has become a major therapeutic objective in the management of Chronic Inflammatory Bowel Diseases (CIBD).

**Objectives:**

To assess the prevalence of sexual dysfunction (SD) in patients with CIBD in remission and compare it to healthy controls (HC), and to determine the associated factors

**Methods:**

This was a cross-sectional study, conducted over 8 months, involving 36 patients with CIBD, who attended the gastroenterology outpatient of Hedi Chaker University Hospital in Sfax (Tunisia). They were compared to 36 HC. Sexual function was assessed with the “Female sexual Function Index” and the “International Index of Erectile Function”.

**Results:**

In the sample of CIBD, the prevalence of SD was 65.4% in women and 50% in men. Compared to controls, patients with male gender had significantly more impaired erection and orgasm (p=0.005; p=0.002 respectively), and those with female gender had significantly more impaired sexual arousal and desire (p=0.003; p=0.028 respectively). In the sample of patients, having a poor marital harmony and a fewer sexual attraction towards partner were correlated with decreased desire (p=0.017) in men and with sexual arousal (p=0.024) and decreased desire (p=0.048) in women. The number of relapses negatively affects erection (p=0.038) and orgasm (p=0.048). Depression correlated with a decreased orgasm (p=0.001) and desire (p=0.048) in men, and with a decreased sexual arousal (p=0.006) in women.

**Conclusions:**

SD is common in CIBD, hence the need for a multidisciplinary approach to allow improvement of the quality of life of these patients, and of their partners.

